# Homologous Recombination as a Replication Fork Escort: Fork-Protection and Recovery

**DOI:** 10.3390/biom3010039

**Published:** 2012-12-27

**Authors:** Audrey Costes, Sarah A. E. Lambert

**Affiliations:** Institut Curie, Centre de Recherche, CNRS, UMR3348, Centre Universitaire, Bat110, 91405, Orsay, France; E-Mail: Audrey.costes@curie.fr

**Keywords:** homologous recombination, DNA replication, DNA repair, fork-restart, fork-repair, fork stabilization

## Abstract

Homologous recombination is a universal mechanism that allows DNA repair and ensures the efficiency of DNA replication. The substrate initiating the process of homologous recombination is a single-stranded DNA that promotes a strand exchange reaction resulting in a genetic exchange that promotes genetic diversity and DNA repair. The molecular mechanisms by which homologous recombination repairs a double-strand break have been extensively studied and are now well characterized. However, the mechanisms by which homologous recombination contribute to DNA replication in eukaryotes remains poorly understood. Studies in bacteria have identified multiple roles for the machinery of homologous recombination at replication forks. Here, we review our understanding of the molecular pathways involving the homologous recombination machinery to support the robustness of DNA replication. In addition to its role in fork-recovery and in rebuilding a functional replication fork apparatus, homologous recombination may also act as a fork-protection mechanism. We discuss that some of the fork-escort functions of homologous recombination might be achieved by loading of the recombination machinery at inactivated forks without a need for a strand exchange step; as well as the consequence of such a model for the stability of eukaryotic genomes.

## 1. Introduction

**Figure 1:**
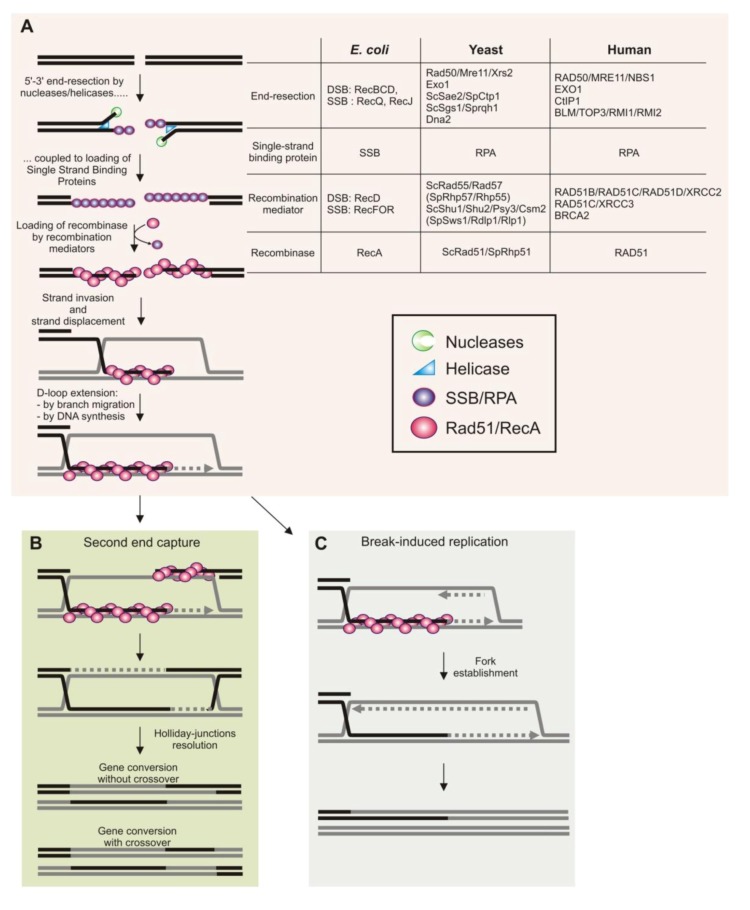
Early Steps of Homologous recombination during double-strand break repair (see text for details). (**A**) Single-Stranded Binding proteins are loaded onto 3'-overhanging single-stranded DNA (ssDNA) resulting from 5'-3' end resection by the concerted action of nucleases and helicases. Recombination mediator proteins help the recombinase Rad51 to nucleate on ssDNA and to form a structured nucleoprotein filament. Recombinases then promote the invasion of the 3' single-stranded end into a DNA duplex, to pair it with the complementary strand, and to displace the non-complementary strand. The resulting three-stranded structure is termed displacement loop (D-loop). The 3' end of the invading strand primes DNA synthesis to copy the DNA template. (**B**) The second extremity of the DSB is captured by annealing of the displaced strand, leading to the formation of double Holliday junctions (dHJs). Repair of the DSB is completed by the resolution of dHJs to form recombinant products that are associated, or not associated, with crossovers (COs, reciprocal exchange of genetic information). (**C**) Model of break-induced replication that can lead to establishment of a replication fork.

The faithful and accurate transmission of the genome through successive cell divisions requires a precise network of pathways coordinating, among others, DNA replication with DNA repair and recombination. Homologous recombination is a mechanism common to all life, necessary for the maintenance of genome stability. Although it is not an essential process in unicellular organisms, it is vital for cell proliferation in metazoans. The final product of homologous recombination mechanism is an exchange of genetic information between DNA molecules. Homologous recombination thus promotes genetic diversity during various biological processes, such as bacterial conjugation, meiosis and gene targeting. Homologous recombination is also an efficient DNA repair mechanism necessary for cell survival when the DNA suffers various types of damage, including double-strand breaks (DSBs), single-stranded DNA gaps (ssDNA gaps) and nicks. The substrate initiating the process of homologous recombination is a single-stranded DNA (ssDNA)molecule coated with single-strand DNA-binding proteins (SSB in prokaryotes and RPA in eukaryotes) (Figure 1). With the assistance of recombination mediator proteins (RMPs), the recombinase protein (RecA family in prokaryotes or Rad51 family in eukaryotes) nucleates onto ssDNA to form a nucleoprotein filament. This RecA/Rad51/ssDNA structure is the competent intermediate for the initiation of genetic exchange between homologous DNA molecules (see Figure 1 for details). The nucleoprotein filament can invade a homologous DNA duplex, pairing the invading ssDNA with the complementary strand of the DNA duplex and displacing the non-complementary strand. The resulting three-stranded structure is called a displacement loop (D-loop, Figure 1). When performed *in vitro* with appropriate RMPs and two homologous DNA duplexes, the strand exchange reaction results in complete exchange of the two complementary strands (referred to as a 4-strand exchange reaction);this is the molecular basis for genetic exchange *in vivo* [1,2].

Any mechanism linked to DNA metabolism which generates ssDNA as an intermediate offers a potential starting point for homologous recombination. Therefore, to avoid superfluous genetic exchange, that is potentially detrimental for the stability of the genome, there is sophisticated regulation of various steps of homologous recombination during the cell cycle and in response to DNA damage [3]. Because of competition with alternative DSB repair as Non Homologous End Joining (NHEJ), regulation of homologous recombination is particularly sophisticated in mammalian cells. Indeed, signaling pathways (for example the Fanc pathway) have key caretaker functions for genome stability by ensuring that homologous recombination acts only on appropriate substrates and only during the appropriate phase of the cell cycle [4]. 

**Figure 2:**
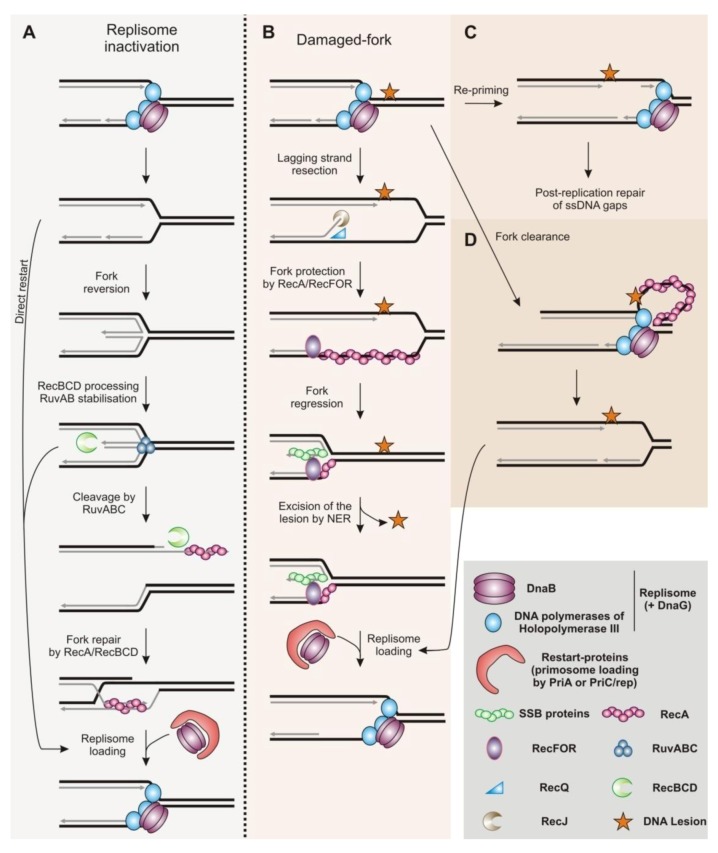
Recombination function at replication forks in Bacteria (see text for details). (**A**) Model of fork reactivation at inactivated forks. (**B**) Model of fork-stabilization at damaged-forks, without a strand exchange step by the recombinase. (**C**) Model of post-replication repair of ssDNA gaps left behind moving forks. (**D**) Model of fork-clearance at damaged-forks, without a strand exchange step by the recombinase.

**Figure 3:**
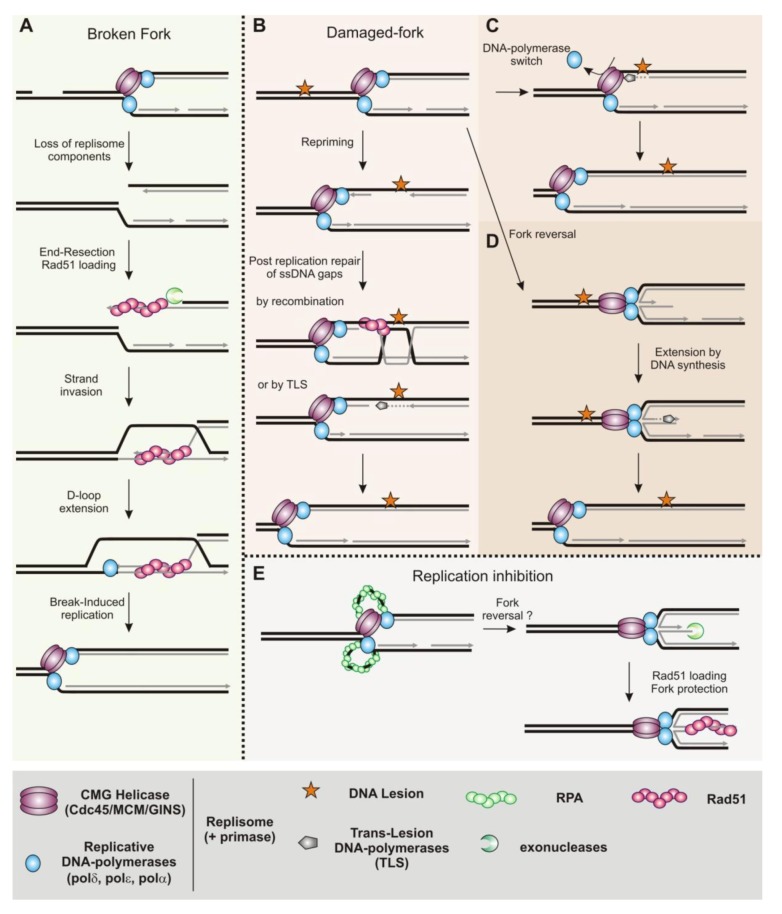
Recombination function at replication forks in eukaryotes (see text for details). (**A**) Model of repair of broken forks. (**B**) Model of post-replication repair of ssDNA gaps left behind moving forks. (**C**) Model of polymerase-switch at damaged-forks (recombination independent). (**D**) Model of fork reversal at damaged forks (the role of recombination in this pathway remains unclear). (**E**) Model of fork-stabilization upon inhibition of the elongation step, without a strand exchange step by the recombinase.

In addition to DSBs repair, homologous recombination is an essential mechanism for robust DNA replication. By promoting recombination between sister chromatids, homologous recombination ensures that any ssDNA gaps left behind moving replication forks are correctly sealed (Figure 2C and Figure 3B). Homologous recombination is also involved in the rescue of replication forks impeded in their progression and in the repair of broken forks. In bacteria, homologous recombination contributes to reassembling a functional replisome at inactivated replication forks [5]. There is various evidence in eukaryotes that homologous recombination is involved in rebuilding replisomes, but details of the mechanism of origin-independent loading of the replisome at inactivated forks have only started to emerge. The mechanisms of genetic exchange during the repair of DSBs have been described by biochemical and molecular studies, whereas the mechanisms by which homologous recombination promotes the resumption of DNA synthesis at inactivated replication forks remain unclear. Recent investigations in human cells and in Xenopus show that homologous recombination has a fork-protection function [6]. The recombinase Rad51 and its recombination mediator partners might act at impeded replication forks to stabilize them by protecting newly synthesized strands from extensive end-resection. This fork-protection function appears genetically separable from the DSB-repair function of homologous recombination. Work in bacteria suggests a role for RecA and its associated recombination mediators in protecting damaged replication forks from extensive degradation by nucleases. Indeed, a multiplicity of recombination functions at halted forks has been described in *E. coli* and homologous recombination is not necessary only for fork reactivation but also acts as a fork-stabilizer until DNA synthesis is resumed [7].In this review, we focus on the support function of homologous recombination for DNA replication, especially at inactivated replication forks. 

## 2. Homologous Recombination during DNA Replication in Bacteria

Efficient repair of inactivated replication forks is vital to bacteria [8]. The genome of bacteria generally consists of a single chromosome, although several bacterial species have a genome divided into several molecules. However, in every case, each chromosome is replicated by a single pair of divergent replication forks initially built at a single origin (*oriC*). For circular chromosomes, the two divergent forks terminate their progression at a single termination region, located opposite to *oriC*. Thus, the duplication of the bacterial genome is a perilous process because neither initiation at alternative origins nor the progression of the opposite fork can rescue a halted replication fork. In cases of fork arrest, DNA replication can only be completed by resumption of the inactivated fork. Consequently, multiple pathways have evolved to ensure efficient restart of arrested forks: this includes removal and/or the repair of the damage responsible for the particular fork arrest, and mechanisms for re-assembling the replication machinery (the replisome) at inactivated forks [9,10]. Impediments to fork progression can arise from various causes and the multiplicity of fork-restart mechanisms corresponds to the need to rescue appropriately forks inactivated in a variety of pathways. In this review, we focus on the functions of homologous recombination at arrested forks in the bacterium *Escherichia coli*(*E. coli*) in which these pathways have been most extensively studied. The restart of inactivated replication forks requires the replisome to be rebuilt; this involves the loading of the replicative helicase DnaB onto the chromosome at a position other than *oriC* and independently of the replication initiator DnaA (Figure 2). To do so, the primosome (composed at least of the replicative helicase DnaBand the primase DnaG) is loaded by restart proteins, and then the holopolymerase III (containing replicative DNA polymerases and theirs co-factors) is recruited to form the replisome. In *E. coli*, restart proteins require a helicase activity to load the primosome at arrested forks: either PriA or the PriC/Rep complex, PriA being the major pathway [10,11]. According to the structure of the arrested fork, it can be either restarted directly by PriA and PriC/Rep pathways, or handled by homologous recombination [12]. Many thermosensitive replication mutants have been studied to investigate the restart of replication forks inactivated following the loss of a functional replisome [5]. The recovery of DNA replication after the fork encounters DNA damage (caused by UV-C radiation) and has also been extensively studied. Investigations of homologous recombination in *E. coli* have revealed the pivotal functions of homologous recombination at inactivated forks, and particular roles have been identified according to the cause of the fork arrest.

### 2.1. Restart of Inactivated Replication Forks by Homologous Recombination

Homologous recombination plays a crucial role in the repair of blocked replication forks [5,8,13,14,15]. At an inactivated replication fork, the homologous recombination machinery remodels the forked structure creating a recombination intermediate recognized by PriA which recruits a replication restart complex; this allows the successive assembly of the primosome and of the replisome such that the fork restarts (Figure 2A and Figure 2B). PriA is able to recognize a D-loop structure and allows the recruitment of the replicative helicase DnaB through its interaction with the restart-proteins PriB/DnaT and the helicase loader DnaC [11,16,17,18,19,20,21].

Recombination mediator proteins (RMPs) aid the nucleation ofRecA onto ssDNA coated by single-stranded DNA binding protein (SSB).SSB proteins play a dual role in the formation of RecA filament: they have a positive action by preventing the formation of secondary structures in ssDNA; however, RecA nucleation is prevented when ssDNA is covered by SSB [22,23]. Consequently, RMPs promote the formation of the RecA nucleofilament, either by facilitating the nucleation of RecA and/or by displacing SSB proteins from ssDNA [24,25,26].The mechanism of RecA loading depends on the type of the substrate. The two major pathways of RecA loading are the RecFOR pathway forsingle-strand break lesions and the RecBCD pathway for double-strand break lesions [27,28,29,30]. Seven genes are involved in the RecFOR pathway: RecA, RecQ, RecJ, RecF, RecO, RecR and RecN. The loading of RecA at ssDNA gaps requires the concerted action of the 3'-5' DNA helicase RecQand the 5'-3' exonuclease RecJ [31,32,33,34]. Then, the RMPs RecF, RecO and RecR form a complex facilitating RecA loading onto the ssDNA. RecFOR can load RecA at the junction between ssDNA-dsDNA, and prevents RecA filament extension on dsDNA beyond the ssDNA gap [35]. At double-strand breaks, the nuclease activities of RecBCD resect blunt-ends extremities, and at Chi sites, RecBCD generates a 3' single-stranded end. RecBCD is also responsible for RecA loading onto ssDNA and helps its nucleation, but is not considered to be a formal RMP protein [28,36].

Replication defects due to the inactivation of one or more components of the replisome can result in chromosomal breakage [37,38,39,40]. It is estimated that around 1% of cells, in a population growing under normal conditions, experience either a double- or a single-strand break due to impediments to replication forks [28,41]. Replication-coupled dsDNA ends are produced either when the fork encounters a nick or a ssDNA gap, or during the processing of inactivated forks [5,28]. Thermosensitive mutants of the holopolymerase III (mutated for a component of the clamp loader HolD, the beta clamp DnaN or the replicative DNA-polymerase DnaE) and mutants of the replicative helicase DnaB require the RecBCD pathway for cell survival at semi-permissive temperature for growth, indicating the occurrence of dsDNA ends [5].Similarly, Pulsed-Field Gel Electrophoresis (PFGE) experiments showed that defects in the holopolymerase III or DnaB lead to chromosomal breakage that results from the activity of the RuvABC complex, the bacterial resolvase [38,40,42]. Loss of a functional replisome at active replication forks leads to the annealing of the two nascent strands, resulting in a 4-branched structure called a reversed fork and resembling a Holliday junction (Figure 2A). The reversed fork is then cleaved by RuvABC, creating a DSB which is resected by RecBCD. The recombinase RecA is then recruited onto ssDNA generated by RecBCD and remodels the fork into a structure recognized by PriA, thus allowing the primosome and then the replisome to be loaded at the inactivated fork [5]. This fork-restart model involving cleavage of the reversed fork by RuvABC applies to other mutations affecting DNA replication, for instance mutations in the accessory helicase Rep and mutations that specifically inactivate PriA helicase activity [42,43]. Alternatively, the dsDNA end of the reversed fork is resected by RecBCD to recruit PriA and the primosome directly, independent of the cleavage of the reversed fork by RuvABC and of RecA recruitment. This last model has also been proposed recently to explain the direct restart of replication forks that collapse following collision with the transcription machinery [44].

The reversal of the fork results either from topological stress, or is actively driven by enzymes. Several proteins have been shown to be able to reverse inactivated forks *in vivo* or *in vitro*: the helicases RuvAB and RecG, the recombinase RecA, and the beta clamp DnaN itself [37,38,40,43,45,46,47,48]. When the processivity of the replisome is compromised (in *dnaE* or *dnaN* mutants), the helicase UvrD favors fork reversal but mainly by promoting the disassembly of the RecA filament formed on ssDNA gaps at inactivated forks. In this case, RecA loading at replication forks requires the RecQJFOR pathway, consistent with RecA being recruited before the formation of DSB [49,50]. *In vitro*, RecA is able to reverse a model forked structure containing assDNA gap on the leading strand. The reaction is stimulated by the presence of SSB proteins, but is inhibited by the presence of the recombination mediator proteins RecFOR [48]. *In vivo*, RecA causes fork reversal upon loss of a functional replicative helicase (in a *dnaB* mutant) and the ability of RecA to reverse the fork is independent of both known RecA loading mechanisms (RecFOR and RecBCD pathways) and independent of the induction of the SOS system [45]. The induction of the SOS system involves the formation of a RecA filament on ssDNA. Possibly, a small amount of RecA bound to ssDNA, below the threshold necessary to induce the SOS response, might be sufficient to reverse the replication fork. Alternatively, there may be a third uncharacterized RecA-loading pathway in *E. coli*. Indeed, both the genetics and the molecular details of replication fork reversal by RecA remain poorly understood. 

Thus, the machinery of homologous recombination is clearly involved in the restart of inactivated replication forks by a mechanism that involves a DSB (RecBCD pathway) resulting from the cleavage of reversed forks. RecA also acts at inactivated forks before the cleavage of the reversed fork, being recruited to ssDNA gaps with the assistance of the RecFOR pathway. In addition, RecA promotes the reversal of inactivated forks by an unknown mechanism. One possible function for the RecA/RecFOR recombination machinery at ssDNA gaps during DNA replication is to protect the inactivated fork from extensive DNA resection. The RecQJFOR pathway has been extensively studied in response to DNA damage and especially to UV-C irradiation. 

### 2.2. Replication Fork Protection by Homologous Recombination

Replication forks are halted by many types of DNA damage and there have been numerous studies in *E. coli* to determine how DNA synthesis proceeds on a damaged template. UV-C irradiation induces predominantly pyrimidine dimers, which are repaired by nucleotide excision repair (NER) involving the excinuclease UvrABC, and has been widely used for such studies [51,52,53,54]. Pyrimidine dimers do not impede the replicative helicase but stop the DNA polymerase. The replisome acts as a molecular motor, catalyzing nucleotide polymerization and unwinding of the DNA template, and pyrimidine dimers may impede the progression of the replication fork and a transient uncoupling between the replicative helicase and DNA polymerase [51,55,56]. The excision of such lesions by the UvrABC pathway generates assDNA gap, and this may also impede the progression of the replication fork [57]. 

The consequences of DNA lesions encountered by the fork depend on the strand affected. It is thought that a blocking lesion on the lagging strand has little impact on the efficiency of DNA synthesis, because the repeated priming for Okazaki fragments allows DNA synthesis to be primed downstream from the lesion. AssDNA gap is then left behind the moving fork and is subsequently filled by the RecA/RecFOR recombination machinery acting in a post-replicative manner [33,58,59,60,61]. The consequences of a blocking lesion on the leading strand remainthe subject of debate. Until recently, the prevailing model was that the replisome cannot bypass a blocking lesion, and thus the resumption of DNA synthesis would require fork-restart mechanisms (either by homologous recombination or by a direct restart via the helicases PriA and PriC/Rep). Blocking lesions on the leading strand may also result in a transient uncoupling of the DNA synthesis of the leading strand from that on the lagging strand [62,63]. However, a recent *in vitro* study showed that the bacterial replisome is tolerant to DNA damage: the replisome is able to re-prime DNA synthesis downstream from a single pyrimidine dimer on the leading strand (Figure 2C). This re-priming on the leading strand does not require fork-restart proteins, including PriA or PriC/Rep, and does not involve dissociation of the replisome [64]. Re-initiation of DNA synthesis downstream from the DNA lesion on the leading strand thus results in a ssDNA gap left behind the moving fork that could be repaired by homologous recombination. Re-priming of this type may be sufficient for efficient DNA synthesis in situations of normal spontaneous DNA damage, but the intrinsic ability of the replisome to replicate on a damaged template might be overwhelmed when cells are exposed to exogenous DNA damage, for example when exposed to UV-C irradiation. 

Upon UV-C-induced DNA damage, DNA synthesis stops and then resumes synchronously after a delay of 15 to 20 minutes [65,66,67,68]. It has been suggested that this delay is the time needed to process impeded forks (*i.e.*, repair of damaged nucleotides and/or restart of replication forks). The inhibition and the resumption of DNA synthesis after irradiation have been studied by following the incorporation of radio-labeled nucleotides post-irradiation. Several proteins are involved in the recovery of DNA replication, including NER proteins, the RecQJFOR recombination machinery, the recombinase RecA, the fork-restart proteins including PriA, and DnaC, the loader of the replicative helicase. Genetic analysis indicates that the resumption of DNA synthesis on a damaged template requires the excision of the lesion, the recruitment of homologous recombination machinery and the re-assembly of a replisome to restart inactivated forks [34,65,66,67,68,69,70,71,72]. The concomitant action of the RecQ helicase and the RecJ exonuclease at damaged replication forks allows the controlled resection of the lagging strand, resulting in RecA loading by RecFOR and regression of the fork (*i.e.*, the two parental strands re-annealed together without annealing of the nascent strands). The regression of the fork allows the DNA lesion to be excised from duplex DNA by the NER. Finally, the reassembly of the replisome triggered by PriA permits the replication fork to restart [7,34,73] (Figure 2B).

Homologous recombination appears to have an important function in addition to restarting inactivated forks and filling ssDNA gaps left behind the moving fork. It stabilizes inactivated forks by protecting nascent strands from extensive resection by RecQJ. The resection of nascent strands has been studied by radio-labeling before exposure to UV-C irradiation. In the absence of RecA or RecFOR, nascent strands at inactivated forks are extensively degraded and replication resumption is compromised [7,59,67,68,69,71]. Thus, the homologous recombination machinery is required to maintain the integrity of the damaged fork until the restart occurs. The resumption of DNA synthesis following UV-C irradiation also requires restart-proteins (PriA, PriB or PriC) and the accessory replicative helicase (Rep). Nascent strands are transiently resected in the absence of restart-proteins, but there is no uncontrolled degradation of inactivated forks as observed in RecFOR mutants [72]. Therefore, the extensive resection of nascent forks cannot be merely the consequence of a defect in restarting the fork; RecA/RecFOR appear to have a specific role in protecting inactivated forks by limiting degradation of nascent strands. However, extensive degradation of nascent forks is observed in cells mutated for *dnaC*, the loader of the replicative helicase DnaB, in which inactivated forks are unable to resume DNA synthesis [68]. The extensive resection of nascent strands in the *dnaC* mutant is dependent on both the RecJ nuclease and the RecBCD pathway, indicating that there are DSBs, or at least dsDNA ends, formed in the absence of DnaC following UV-C irradiation. Thus, cells mutated for *dnaC* suffer from problems in addition to defects in restarting replication forks halted byDNA lesions.

In the restart model proposed by J.Courcelle, and reinforced by other groups, homologous recombination has two functions at forks halted by DNA lesions: it facilitates the removal of the DNA lesion, and it has a fork-protection function (Figure 2B). Nonetheless, it is unclear whether excision of the lesion by the NER during DNA replication requires regression of the fork and its stabilization by the RecA/RecFOR machinery. Also, an interesting issue is whether the fork-protection function is necessary and sufficient for efficient restart of inactivated forks. It has been suggested that the RecA/RecFOR pathway acts at damage-induced halted forks to dislodge the holopolymerase III blocked by a DNA lesion on the leading strand [74]. The driving force of the RecA filament may be strong enough to allow protein-mediated displacement, such that RecA has a previously undescribed fork-clearance function (Figure 2D). While the fork-restart function of the homologous recombination machinery, especially when a DSB is involved, is mechanistically well understood, the fork-protection and the fork-clearance functions of RecA/RecFOR remain poorly characterized. It is also unclear whether these two functions are important for the resumption of DNA synthesis on a damaged template. The ability of RecA to restart a broken replication fork or to reverse an inactivated fork requires its ability to promote strand invasion and exchange. By contrast, the loading of RecA by RecFOR at ssDNA gaps might be sufficient for fork-protection and fork-clearance functions, without the need for a strand invasion step as previously suggested by J. Courcelle [75,76]. In this case, the fork-stabilization function by the recombination machinery might refer to a strand exchange-free mechanism (SEX-free fork-escort function). However, there is no direct evidence that the fork-stabilization function by recombination enzymes is independent of a strand invasion step. 

Interestingly, an old but intriguing RecA mutant has been characterized as a potential separation-of-function mutant: the *recA433* mutant contains a single mutation leading to an arginine to histidine substitution at residue 243, located in the core region conserved in the Rad51-family homologue [77]. RecA^R243H^ supports conjugational and transductional recombination and the induction of the SOS system, suggesting that the mutated protein is able to bind ssDNA and to promote a strand exchange reaction. However, RecA^R243H^ is defective for resumption of DNA synthesis, resulting in a high sensitivity to UV-C. In the *recA^R243H^* mutant there is extensive degradation of nascent forks as in the absence of RecA/RecFOR [77]. Trans-lesion synthesis is in part compromised in *recA^R243H^*, but this defect is unlikely to explain the inability of the mutant to resume DNA replication after UV-C irradiation, because trans-lesion synthesis is not essential for resumption of DNA replication on a damaged template [66]. Thus, the single amino acid substitution in RecA^R243H^ seems to specifically affect RecA function linked to the RecFOR pathway, but not the function linked to the RecBCD pathway. The details of the loss of function (either in protein-protein interactions or in biochemical properties) associated with the substitution in RecA^R243H^ are unknown, but these observations suggest that the functions of RecA in restarting broken replication forks and in fork-clearance/stabilization are genetically separable. 

### 2.3. Homologous Recombination as an Escort of Fork Progression

Over the last decade, cell imaging based on the use of fluorescent-protein fusions has contributed to elucidating the dynamics of fork-restart and repair mechanisms. The emerging view is that some components of the fork-restart machineries, including proteins of the homologous recombination pathway, are associated with the moving replication fork, whereas other factors, including the recombinase RecA, are recruited only once the fork has encountered a DNA lesion. In *E. coli*, RecA forms up to five foci in 10%–15% of a cell population growing under normal laboratory conditions. RecA foci are thought to correspond to sites of RecA nucleofilament bound to ssDNA. Following UV-C irradiation, RecA localizes at the cell center in most cells, suggesting that it is recruited by replication factories [78]. Similar observations were made in *Bacillus subtilis* (*B. subtilis*), in which establishment of a replication fork is necessary forRecA foci to form following DNA damage [79]. RecA focus assembly results from the redistribution of existing RecA molecules in the cell to the site of a halted replication fork, supporting the view that RecA is not pre-associated with the replication machinery, but is recruited at halted forks. By contrast, other recombination factors, particularly RecO, RecQ and RecJ, and restart-proteins, including PriA, are associated with the moving replication fork through their interaction with the SSB protein [80,81]. Thus, the replication fork appears to have a substantial escort of enzymes involved in DNA metabolism during its progression; this escort is thus available to deal with stretches of ssDNA. These fork-escort pathways include some component of the homologous recombination machinery, but not the recombinase itself. The SSB protein, with its interactome, might have a pivotal function in the orchestration of the appropriate response to fork-arrest according to the initial cause of the impediment to fork progression. 

## 3. Homologous Recombination during DNA Replication in Eukaryotes

In eukaryotes, DNA replication of linear chromosomes is initiated at multiple replication origins that are activated with different efficiencies, according to a spatio-temporal program [82,83,84]. In *S. cerevisiae*, each origin is characterized by a defined efficiency and a related timing of firing. In mammalian cells, the firing of replication origins is more stochastic and origins are clustered into replication domains of 400–800 Kb that are replicated either early or late. Once activated, a replication origin gives birth to two replication forks that progress in bi-directional directions until they merge with a converging fork at a replication termination site, such that DNA synthesis of the replicon is completed. Replication termination in eukaryotes is thought to occur randomly. However, unlike replication initiation that has been extensively studied, little is known about how eukaryotic cells orchestrate DNA synthesis during fusion of converging forks. Once established, a single replication fork will replicate several tens of thousands of bases before meeting a converging fork. The fork does not progress at a constant rate and many obstacles slow transiently or robustly arrest fork movements. Non-histone proteins tightly bound to DNA, structure-forming sequences, conflict with DNA metabolic processes including transcription, chromatin organization (at repressed genes for example) as well as DNA damage and are all liable to impede fork progression [85,86,87].

A single halted fork does not necessarily prevent the completion of DNA replication because a fork progressing in the opposite direction from an adjacent origin will replicate up to the site of the fork arrest. Thus, unlike bacterial chromosome replication, where the rescue of impeded forks byfork-restart mechanisms is essential, activation of dormant origins in eukaryotes can complete chromosomal replication following fork arrest [88,89,90,91]. Indeed, eukaryotic genomes contain more origins than are needed to replicate the genome. Forks traveling long distances might be at greater risk of accident and therefore fork-restart mechanisms might be essential in regions poor in origins, like human fragile sites. The same applies to regions with unidirectional replication (as the ribosomal DNA locus, see below), or when two converging forks are impeded [92,93]. Hindrances to fork progression are factors for replisome malfunction, induction of recombination and genetic instability in yeast models. Therefore, as in bacteria, completion of DNA replication requires replication forks to be well-escorted to ensure their stability, reactivation and merging with converging forks. A multiplicity of fork-restart pathways has been described in yeast models and mammalian cells; both the DNA replication checkpoint and homologous recombination are pivotal mechanisms in escorting the progression of replication forks. Defects in homologous recombination pathways result in a decreased fork velocity, evidence that the recombination machinery acts as a fork-escort mechanism favoring robust DNA replication [94]. The integrity of replication forks is guaranteed by the DNA replication checkpoint that maintains DNA polymerases at the site of nucleotide incorporation to keep the replisomein a replication-competent state [95,96,97]. There is still debate about how the DNA replication checkpoint modulates replisome activities to maintain it in a functional state at halted forks. Nonetheless, the DNA replication checkpoint also regulates nuclease activities (such as Exo1, Mus81 and Dna2) to preserve the integrity of forked structures [98,99,100,101]. In cases of replisome malfunction (referred to as collapsed forks in the literature) or loss of components of the replisome at broken forks, the replisomemust be rebuilt for DNA synthesis to resume (Figure 3A). No PriA or PriC homologues (or putative genes) have been identified in eukaryotes and the loading of components of the replisomeat sites other than replication origins is poorly documented. Nonetheless, in addition to sealing ssDNA gaps during DNA replication, the homologous recombination machinery in eukaryotes appears able to rebuild a replisome at inactivated forks. A fork-stabilizer function for the homologous recombination machinery has also recently been evidenced. 

### 3.1. Recombination Mediator Proteins in Eukaryotes

As in bacteria, the nucleation of Rad51 onto RPA-coated ssDNAin eukaryotes requires RMP activities. In *E. coli*, RecFOR and RecBCD define two distinct pathways for RecA loading onto ssDNA gaps and dsDNA ends, respectively. In eukaryotes, Rad51 loading onto distinct substrates has not been so clearly established genetically and biochemically. Eukaryotic RMPs were recently reviewed in detail by Kreijci *et al.* so we focus here on the main activities of RMPs and their potential function in distinguishing between recombination substrates [3].

In yeasts, the main RMP is the Rad52 family protein (Rad52 in *S. cerevisiae* and Rad22 in *S. pombe*). Rad52 binds both RPA and Rad51, and helps Rad51 nucleation by displacing RPA from ssDNA [3]. Rad52 alsohas a ssDNA annealing activity which serves as a recombination function independently of Rad51, for example to capture the second DNA end during the repair of DSB(Figure 1B) [102,103]. In mammalian cells, the pivotal RMP is BRCA2: loss of BRCA2 function predisposes to breast and ovarian cancer. BRCA2 binds Rad51 and facilitates Rad51 nucleation onto ssDNA by inhibiting its ssDNA-dependent ATPase activity [104,105]. BRCA2 stabilizes the nascent filament by blocking turnover of Rad51 from the DNA and thereby stimulating the strand exchange reaction. It is unclear whether the ability of BRCA2 to displace RPA from ssDNA is required for promoting Rad51 nucleation. Rad51 proteins have more affinity for dsDNA than does RecA, and a key function of BRCA2 is to prevent Rad51 nucleation onto dsDNA beyond the junction between ssDNA and dsDNA. Human cells also express a Rad52 homologue which has a strand annealing activity, but its RMP function is poorly documented and it seems to have only a minor role in Rad51 nucleation [105]. Human Rad52 seems to make a larger contribution to homologous recombination mechanisms when DNA replication is compromised than during the repair of DSB, suggesting that in humans distinct ssDNA substrates might require different RMPs [106]. 

In addition to Rad52 and BRCA2, RMP complexes containing paralogues of Rad51 have been identified from their homology with Rad51, and genetically as factors of homologous recombination both in yeast and human cells. For example, the yeast heterodimer Rad55/Rad57 binds Rad51 and helps to stabilize the Rad51 filament [3,107,108]. In mammals, two RAD51 paralogue complexes serve as RMPs: one composed of RAD51B/RAD51C/RAD51D/XRCC2 and the other RAD51C/XRCC3. The related RAD51D protein in *C. elegans*, RFS-1, is not essential for the repair of DSBs or broken replication forks but is required for Rad51 recruitment at ssDNA gaps induced by UV-C treatment or at forks halted by inter-cross-links or torsional strains [109]. RFS-1 may have a specific function in promoting recombination events at ssDNA gaps rather than at DSBs. Another identified RMP is the Shu complex, composed of Shu1/Shu2/Psy3/Csm2 in *S. cerevisiae* and of Sws1/Rdlp1/Rlp1 in *S. pombe*; this complex is probably conserved in mammals. The biochemical functions of the Shu complex are not well understood. Genetic studies indicate that loss of this complex leads to sensitivity to replication blocking agents but not to DSB-inducing agents; therefore the Shu complex may have a specific function in facilitating replication-induced homologous recombination [110,111,112,113]. Similarly, it has been suggested that the fission yeast Shu complex has a recombination mediator function dedicated to Rad51 loading onto ssDNA gaps [114].

Another strategy used by eukaryotes to discriminate between different recombination substrates is the regulation of homologous recombination by negative regulators and by post-translational modifications, especially phosphorylation and SUMOylation, of RMPs (see review [3] for an extensive list of post-translational modifications).Of particular interest is the anti-recombinase helicase Srs2 involved in the disassembly of Rad51 filaments from ssDNA. The RMP complex Rad55/Rad57 forms a co-filament with Rad51 and the resulting filament is more resistant than the Rad51 filament itself to dissociation by Srs2 [115]. Thus, the RMPs used to stabilize Rad51-filament might influence its fate. In addition, Srs2 interacts with PCNA, the clamp loader of replicative DNA polymerases, an interaction that requires the SUMOylation of PCNA [116,117]. The SUMOylated form of PCNA may allow the recruitment of Srs2 at replication forks where the anti-recombinase prevents deleterious recombination events.Rad55 is phosphorylated on serines 2, 8 and 14by the checkpoint kinase Rad53 in response to DNA damage and replication inhibition [118,119].This may enhance the recombination mediator function of Rad55. AnunphosphorylableRad55 mutant exhibits defects in the recovery of DNA damage-induced halted forks, but remains proficient in promoting repair of chromosomal DSBs and of gapped plasmids by homologous recombination [119]. These data suggest that phosphorylation of Rad55 affect its discrimination between different recombination substrates: ssDNA gaps at halted forks and ssDNA extremities. Understanding how Rad55 phosphorylation affects the activity of Srs2 at halted forks will provide important clues about the regulation of Rad51 loading during repair of replication-associated DNA lesions. 

### 3.2. Homologous Recombination Is Coupled to DNA Replication

In mammalian cells, *in situ* immunofluorescence-based studies have shown that Rad51 and BRCA2 form punctuate nuclear foci during S-phase. Following DNA damage or replication inhibition, Rad51 and BRCA2 co-localize within foci at sites of ssDNA that also contain RPA [120,121,122,123,124]. These Rad51 foci are called recombinational centers, because they are believed to be the sites of DNA repair by recombination. Recombination foci are preferentially associated with the replicative chromatin, consistent with the homologous recombination machinery acting during DNA replication and as a post-replication repair mechanism [125]. A subset of S-phase specific or damage-induced recombination foci also contains the replication factor PCNA, suggesting that recombination centers are also sites of DNA synthesis [126,127,128]. The association of Rad51-foci with a replication factor during S-phase led to the view that homologous recombination can be coupled to DNA replication. Recombination centers were then identified in yeast models and their dynamics investigated with fluorescently tagged proteins [107,129]. Genetic studies and real-time cell imaging indicate that recombination foci are sites of DNA repair, but it remains unclear whether each recombination repair event gives rise to a single recombination focus. Further evidence for a link between DNA replication and homologous recombination is that spontaneous Rad52 foci form mainly during S-phase in budding yeast and that defects in the DNA polymerase alpha increase the numbers of recombination foci in S-phase [130]. Work with conditional site-specific fork arrests in fission yeast showed that Rad22, the homologue of Rad52, is recruited at blocked forks to ensure their restart, establishing a direct connection between homologous recombination and DNA replication in eukaryotes [131,132,133].

Some of the first evidence of a physical link between homologous recombination and DNA replication was from a study in budding yeast by Rothstein's lab in 1997. Zou and Rothstein identified recombination structures resembling Holliday junctions at the ribosomal DNA (rDNA)array in *S. cerevisiae* [134]. The rDNA locus is organized as 100-200 direct repeats, each repeat containing the sequences encoding the 35S rRNA transcribed by RNA-polymerase I, the 5S rRNA transcribed by RNA polymerase III and a replication origin (ARS). To avoid head-on collisions between the transcription machinery of RNA-polymerase I and the replication fork apparatus, there is a polar replication fork barrier (RFB) within the non-transcribed region. This RFB blocks the progression of the replication fork moving in the opposite direction to the transcription bubble [135]. Such RFBs are potential substrates for homologous recombination. Zou and Rothstein therefore exploited the rDNA array for the analysis of the formation of potential recombination structures(or joint-molecules, JMs) using two-dimensional gel electrophoresis (2DGE). Defects in the DNA polymerases alpha or delta, but not epsilon, and thus in the DNA synthesis of the lagging strand, resulted in the accumulation of JMs during the replication of the rDNA. Surprisingly, accumulation of JMs was dependent on the main RMP Rad52 but not on the recombinase Rad51. Although it was not demonstrated in this study that accumulation of JMs was linked to the active RFB, this work provided experimental evidence for homologous recombination being active during DNA replication. It also supports the view that accumulation of ssDNA gaps on the lagging strand stimulates homologous recombination [134].

A recent study has revealed in detail the contribution of the homologous recombination machinery to repairing DSB in S-phase [136]. In human cells, DSBs are repaired either by homologous recombination or by non-homologous end joining (NHEJ). NHEJ consists of the ligation of the two dsDNA ends at the break, a process that is mainly error-free. In view of the relative radiation sensitivities of cell lines defective for either homologous recombination or NHEJ, it was thought that DSBs are mainly repaired by NEHJ in mammals, in contrast to what is observed in yeast models. By analyzing recombination and NHEJ centers, Karanam and co-authors showed that NHEJ is indeed the major DSB repair pathway in G1 and G2 cells, whereas homologous recombination becomes gradually more important for the repair of DSBs as DNA replication activity increases [136]. 

The connection between DNA replication and homologous recombination is now well established and, as in bacteria, the homologous recombination machinery probably serves as a fork-escort to ensure complete and faithful genome replication. Although several checkpoint mediators involved in fork stabilization escort the replication fork through physical interactions with components of the replisome, the potential interactions between recombination factors and the replication machinery remain poorly understood. Human Rad51 interacts with RPA and the N-terminal part of BRCA2 was reported to interact with RPA but this interaction was not confirmed by tests with the full-length BRCA2 [105,122,137]. Nonetheless, direct interaction between a RMP and the eukaryotic single-stranded DNA binding protein would support the view that homologous recombination proteins act as fork-escorts, available if there is an impediment to fork progression. The first interaction between recombination and replication factors was described by Shukla *et al.* They reported that purified human Rad51 and Rad52 interact with the MCM (Mini Chromosome Maintenance) complex, a component of the replicative helicase [138]. In fission yeast and human cells, interactions have also been described between Rad51 and MCM (MCM4 interacting with Rhp51 in *S. pombe* and MCM7 interacting with Rad51 in Hela cells) [139]. These interactions occur either during normal S-phase or when DNA replication is slowed by treatment with replication inhibitors. It has been suggested that interaction between the recombinase Rad51 and the MCM helicase promotes the recovery of stalled forks, but the significance of these interactions remains to be established. Finally, in *Trypanosome brucei*, the essential replication factor Cdc45 interacts with the BRCA2 orthologue [140]. However, there is no evidence either in yeast or human cells that homologous recombination factors are associated with replication factories during normal cell growth. The current view is rather that homologous recombination enzymes are recruited at replication forks once their progression is impeded. It is unclear whether protein-protein interactions described above contribute to the recruitment of the homologous recombination machinery at the site of halted forks, or contribute to the recruitment of replication factors during rescue of halted forks by recombination to rebuild a replisome (see below). 

### 3.3. Post-Replication Repair by Homologous Recombination

Homologous recombination efficiently sea lsssDNA gaps behind moving forks that have encountered DNA lesions(Figure 3B). Analysis of DNA fibers in budding yeast treated with methyl methane sulfonate (MMS, a base alkylating agent)shows that ssDNA gaps accumulate in the absence of Rad51, and therefore that the homologous recombination machinery is required to complete DNA replication when the parental DNA is damaged [141]. This damage tolerance pathway implies that there is a re-priming event at the blocked leading strand. Analysis of purified damaged replication forks *in vivo* by electronic microscopy have shown that ssDNA gaps accumulate both just at and behind the moving fork on both sister-chromatids, especially in the absence of post-replication repair pathways, including homologous recombination [142,143]. These observations, both in budding yeast and Xenopus, support the existence of re-priming of the leading strand in eukaryotes, but the tolerance of eukaryotic replisome to DNA damage remains to be explored (Figure 3B–3D).

To seal ssDNA gaps, the homologous recombination process copies the information from the undamaged sister-chromatid. This mechanism, often called template switching in the literature, is expected to involve the formation of recombination intermediates between sister-chromatids. Using 2DGE, joint-molecules formed between sister-chromatids have been identified in S-phase cells replicating parental DNA damaged by MMS treatment [144,145]. Although the exact nature of these joint-molecules is not clearly understood, genetic analyses indicate that they correspond to recombination intermediates resulting from a strand exchange reaction driven by Rad51 between an intact and a gapped sister-chromatid behind replication forks. Sister-chromatid joint-molecules are believed to be resolved by the concerted action of the helicase Sgs1 and its partner topoisomerase 3 [146,147]. The mechanisms involved in post-replication repair of ssDNA gaps following UV-C irradiation and in the formation of sister-chromatid junctions have a common genetic requirement; this is also consistent with joint-molecules being formed behind moving forks and not at halted fork *sperse* [148]. RPA, the RMPs Rad52 and Rad55/Rad57, the 5'-3' nuclease Exo1 and the replicative DNA polymerase delta, but not epsilon, are all required for the formation of sister-chromatid junctions during the replication of damaged templates. Thus, as described in bacteria, it is possible that ssDNA gaps have to be firstly resected to recruit RMPs and to nucleate Rad51 on ssDNA gaps [149]. Following strand invasion (either by the Rad51-coated ssDNA gap itself or by the 3' end of the newly synthesized strand of the gapped sister-chromatid), DNA polymerase delta and its accessory subunit Pol32 may then seal the gap by extending the 3' end. As stated above, although basic recombination mechanisms are conserved between bacteria and eukaryotes, there are differences in the signaling and the regulation of recombination processes. Indeed, post-replicative homologous recombination in eukaryotes is tightly regulated by the poly-ubiquitination and SUMOylation of PCNA [145,148]. However, it remains unclear if post-translational modifications of PCNA act as positive regulators by ensuring that homologous recombination enzymes are recruited to the right substrate at the right time, or if they act as negative regulators, for example by regulating the anti-recombinase helicase Srs2 to avoid undesirable recombination processes at replication forks.

### 3.4. Replication Fork Restart and Repair by Homologous Recombination

Homologous recombination is essential for restarting replication forks both in yeast models and mammalian cells. Fork passage over a ssDNA nick or gap in the parental DNA results in a broken fork with one sister chromatid becoming physically detached from the forked structure(Figure 3A). Such broken forks are efficiently repaired by the homologous recombination machinery [150,151,152,153]. Inter-strand cross-link (ICL) is another kind of DNA damage that impedes the progression of the fork, by preventing unwinding of parental DNA ahead of the fork. The ICL at arrested forks are cleaved by endonucleases, resulting in a broken replication fork repaired by homologous recombination [154].

There is evidence that fork can be restarted by homologous recombination independently of DSBs, and that DSBs at replication forks are not essential for recruitment of recombination factors [131,132,133,143,155,156,157]. In yeast models, recruitment of recombination factors at halted forks appears to be linked to replisome malfunction. Indeed, replication fork stalling due to hydroxyurea treatment (an inhibitor of the ribonucleotide reductase leading to defective bulk synthesis of dNTP during S-phase) is not sufficient for recombination factors to be recruited [107,158]. By contrast, disruption of the DNA replication checkpoint, which leads to replisome dysfunction at the site of nucleotide incorporation, leads to recruitment of recombination factors at stalled forks. More precisely, homologous recombination appears to be actively repressed upon inhibition of DNA synthesis by HU treatment, and this repression by the Mrc1-branch of the DNA replication checkpoint limitsend-resection [141]. These data suggest that, at least in yeast models, recruitment of the homologous recombination machinery at stalled forks is undesirable while the replisome remains functional. This contrasts with the situation in mammals in which controlled exposure to HU induces the association of Rad51 with replicative chromatin [159,160]. In mammals, prolonged treatments with HU lead to replicative DSBs, a phenomenon that is observed only in the absence of the checkpoint pathway in yeast models [99,161,162,163]. HU-induced DSBs are thought to result from inactivation of replication forks followed by their breakage, a process also regulated by the DNA replication checkpoint and the endonuclease Mus81 [99,100,159,163]. It has been suggested that mammalian Rad51 first protects stalled forks and then repairs broken replication forks by mechanisms that are mechanistically and temporally distinct and separated [160].

In addition to repairing broken replication forks, the homologous recombination process is also required to rebuild replisomesat inactivated forks to allow DNA synthesis to resume. Genetic analysis of the break-induced replication (BIR) mechanism has improved our understanding of the ability of homologous recombination to build a replisome during repair of DSBs (Figure 1C and Figure 3A). BIR efficiently repairs DSBs at which the homology with the donor molecule is limited to one end of the DSB. After strand invasion, extension of the 3' invaded-end leads to DNA synthesis over hundreds of kilo bases and can thereby restore the duplication of a full chromosome arm [164,165]. Because long stretches of DNA can be synthesized during BIR, it has been suggested that BIR may be able to repair a broken fork thus ensuring completion of DNA replication, and maintaining telomere length in the absence of telomerase [166]. BIR has mainly been studied in cells arrested in G2, although recent work supports the view that BIR can indeed act during DNA replication to repair forks encountering a single strand nick [150]. BIR requires homologous recombination factors—including the main RMP Rad52, the recombinase Rad51 and the RMPs Rad55/Rad57—and the leading and lagging strand apparatus. The DNA polymerases alpha and delta are necessary for the initial extension step, and the DNA polymerase epsilon is required subsequently, only after the first 30 Kb of DNA has been synthesized [167]. These observations suggest that the establishment of a fully functional replication fork apparatus at DSBs requires time after the initial steps and thus maturation. BIR requires all components of the replicative helicase (Cdc45, the MCM2-7and GINS complexes, the CMG helicase) but replication factors (ORC and cdc6) involved in pre-replication complex (Pre-RC) assembly at replication origins are not essential for DNA synthesis during BIR [168]. How replicative helicase is loaded onto DNA other than at replication origins remains unclear, but all factors necessary for the recruitment of the CMG helicase (except Cdc6), the initial unwinding of DNA and the initial elongation step are also necessary for efficient DNA synthesis during BIR (Cdt1, Cdc7, Dpb11, Sld3, Mcm10 and Ctf4). Thus, BIR appears to be a mechanism for replication apparatus assembly away from replication origins. There are differences between replication forks built through BIR and replisomes assembled at replication origins: DNA synthesis associated with BIR requires Pol32, a subunit of the DNA polymerase delta, which is not required for conventional DNA replication [167]. More surprisingly, BIR results in the progression of a mutagenic fork, contrasting with canonical replication forks that are very much more error-free [169]. In fission yeast, fork shalted by a protein tightly bound to DNA are restarted by homologous recombination factors through a mechanism independent of a DSB. These restarted forks are also mutagenic and prone to replication slippage and template switches [170,171]. Therefore, the process of homologous recombination allows the assembly of a replisome, sufficiently functional to ensure synthesis of hundreds of kilo bases of DNA, but not accurate DNA synthesis. 

The dynamics of loss and reloading of replisome components at broken replication forks have been recently investigated in Xenopus [152]. Upon fork passage through a nick, only a subset of components of the fork apparatus is lost, challenging the view that the replisome needs to be completely rebuilt during the repair of broken forks. The GINS subunit (SLD5 and PSF2), and the DNA polymerase epsilon, but not alpha, are uncoupled from the forked structure. Their re-loading requires the repair of the broken fork by recombination mediated by Rad51 and Mre11. Mre11 expresses the nuclease activity of the Mre11/Rad50/NBS1 complex essential for end-resection at DSBs [172]. Surprisingly, repair of the fork also leads to the recruitment of the trans-lesion-synthesis (TLS) DNA polymerase eta [152]. It is unclear whether this DNA polymerase is strictly required to initiate DNA synthesis after the strand invasion step or if its recruitment simply reveals that the restarted replisome has components not present in replisomes built at replication origins. 

The mechanisms by which recombination promotes the loading of a new replisomein eukaryotes have not been fully elucidated and many questions remain. Components of the fork apparatus itself appear to be involved: two mutated alleles of PCNA (pol30–89 carrying F248A and F249A substitutions and pol30–92 carrying a R80A substitution) are defective for supporting BIR [168]. These two PCNA mutations are associated with impaired recruitment of Rad51 and the DNA polymerase eta at broken forks in Xenopus [152]. These data suggest that PCNA has an early function in fork repair by recombination, and not solely a function later during replisome rebuilding after the strand-invasion step.

### 3.5. A Fork-Protection Function for the Homologous Recombination Machinery

The restart of replication forks provides the first clues to understanding the fork-escort function of the homologous recombination machinery. Recent investigations provide new insights by revealing a fork-stabilizer function for recombination proteins and how this function contributes to the robustness of replication fork progression. The first description of the role of BRCA2 in stabilizing halted replication forks came from the lab of Venkitaraman in 2003 [173]. In this study, the stability of unidirectional replication forks in the rDNA region in embryonic murine fibroblasts was examined by 2DGE. Under normal replication condition, fork progression was not affected by the absence of BRCA2. However, inhibition of DNA replication by HU treatment led to forks being unstable and liable to breakage in the absence of BRCA2. Despite an analysis of the incorporation of radio-labeled nucleotides, the authors were unable to detect there section of stalled forks, and the occurrence of neither localized nor limited resections could be excluded. Thus, a fork-stabilizer function for BRCA2 was proposed but without mechanistic explanation.

The fork-stabilizer function has been studied at the molecular level. Schlacher and co-authors used DNA fiber techniques to analyze the dynamics of DNA synthesis upon inhibition and resumption of the elongation step. Newly replicated DNA at the fork, synthesized before the inhibition of DNA synthesis, is extensively resected, at a rate of 1.8 Kb/hour, in the absence of BRCA2 or Rad51 [174]. Resection of the nascent strands is dependent on Mre11, but the nuclease activity itself was not shown to be directly involved; it is therefore possible that MRN regulates the activity of alternative nucleases at stalled forks. BRCA2 protects the fork by stabilizing the Rad51 filament, a function connected to the Fanconi anemia (Fanc) pathway [175]. These findings show that the homologous recombination machinery, in addition to repairing replicative-DNA lesions, has a specific function in protecting the integrity of nascent strands at halted replication forks.

It has been suggested that Rad51 protects nascent strands in Xenopus [143]. Direct visualization of purified *in vivo* forked structures by electron microscopy shows that ssDNA gaps accumulate at replication forks upon Rad51 depletion: gaps are observed behind the moving fork and at the three-way branched structure of the fork. ssDNA gaps behind moving forks result from the activity of the MRN complex, involved in end-resection at DSBs. End-resection and homologous recombination compete with each other during DSB repair [176]. Thus, ssDNA gaps behind the moving fork are likely to result from defects in sealing by recombination and allow extensive resection by MRN. More surprising are the ssDNA gaps at the fork that are not dependent on the MRN complex; they may reflect the involvement of the recombination machinery in preventing uncoupling between leading and lagging strand synthesis. Even in the absence of exogenous DNA damage, 50% of replication forks contain ssDNA of at least 200 nt at the forked structure when Rad51 is depleted. How homologous recombination maintains the coupling between leading and lagging strand synthesis is unclear. Nevertheless, these observations support the view that the recombination machinery escorts fork progression to ensure robust DNA replication, and provide hints about themolecular basis for the decreased fork speed in recombination-defective cells [94]. 

How important is the fork-stabilizer function of homologous recombination? Replication-associated DSBs are often observed in cells defective for homologous recombination. The initial view was that recombination is required to repair spontaneous DSBs associated with the process of DNA replication [177]. However, incomplete DNA replication due to accumulation of halted forks might result in secondary DNA damage as DSB during the next round of replication [178].Thus, homologous recombination proteins may prevent DNA breaks by stabilizing replication forks, rather thanmerely repairing them. 

Stabilization of halted replication forks may be important for the completion of DNA replication. In budding yeast, replication termination zones (TER), which are binding sites for the topoisomerase 2 (Top2), overlap with elements that impedes fork movements [179]. Among the 71 TERs identified, 67 contain one or more fork-pausing elements and 55 are also binding sites for Top2. Top2 is involved in many DNA transactions during S-phase, and mediates topological transitions at replication forks to ensure their movement and stability. Top2 also facilitates the merging of converging forks that could potentially interfere with each other [180]. The observation that TERs overlap both with known RFBs and Top2-binding sites suggests that fork pausing is relevant to the fusion of converging forks, with the movement of one incoming fork being slowed to provide a suitable topology for forks to merge at termination sites. Thus, stabilizing a halted replication fork might facilitate its merging with a converging fork. Note that in fission yeast, homologous recombination restarts forks blocked by a polar RFB mediated by DNA-bound protein. In the absence of recombination, fork restart is impaired and merging of converging forks has been observed by 2DGE. However, this resulted in cell death, showing that the progression of the opposite fork was not sufficient to rescue the defect in fork restart [131,132]. One possible explanation is that recombination stabilizes the impeded fork to maintainit in a structure allowing its merging with the opposite fork. It is also relevant that termination sites, when not correctly processed, are hot spots of recombination [181].

### 3.6. A Possible Separation of Function

Several studies have tried to uncouple the function of recombination in supporting robustness of DNA replication from its DNA repair function. For example, in *Ustilagomaydis*, distinct domains of Brh2, the orthologue of BRCA2, are involved in the regulation of homologous recombination; the role of Brh2 in promoting recombination in response to UV-C irradiation can be uncoupled from its role in promoting recombination in response to replication inhibition [182].

The DSB-repair and the fork stabilizer functions of the homologous recombination machinery are genetically separable. BRCA2 contains several functional domains: a domain containing BRC repeats and binding to Rad51, a DNA-binding domain (DBD) and a C-terminal part also interacting with Rad51. The contribution of these domains to the DSB repair and the fork stabilization functions of BRCA2 have been investigated [174,175]. The DBD domain is neither required for DSB repair nor for fork-stabilization functions. It appears that the ability of BRCA2 to load Rad51 onto ssDNA is sufficient to promote DSB repair but not to stabilize replication forks. By contrast, Rad51-filament stabilization by the BRCA2 C-terminal domain is not required for DSB repair but is required to protect stalled forks. The overexpression of a Rad51 mutant defective for its ATPase activity (K133R) is sufficient to protect nascent strands from end-resection in the absence of BRCA2. Therefore, BRCA2 may stabilize Rad51 filaments at stalled forks by regulating its ATPase activity. The stability of the Rad51 filament, guaranteed by BRCA2, is thus essential for fork stabilization but not for DSB repair functions. The mechanisms by which BRCA2 ensures efficient repair of a DSB are thus different from the mechanisms by which it protects nascent forks from degradation. The fork stabilizer properties of Rad51 and BRCA2 do not involve the RMP Rad54. The exact recombination mediator functions of Rad54 are not clear, although it plays a crucial role after the strand invasion step during the repair of DSB (post-synaptic function) [183]. In mammals, it is possible to uncouple the essential function of Rad51 in cell proliferation from its function in promoting strand exchange to repair DNA lesions. Indeed, dominant negative forms of Rad51 have been identified that strongly inhibit spontaneous, DNA-damage induced and replication-associated recombination events, without affecting the ability of cells to proliferate [184].

Because the DSB repair function and the fork-stabilizer function of homologous recombination are genetically separable, it is possible, as proposed in bacteria, that fork-stabilization by recombination proteins refers to a strand exchange-free mechanism (SEX-free fork escort function). The use of the recombination machinery to recover or stabilize inactivated forks without a strand exchange step being engaged might support the robustness of DNA replication while limiting deleterious non allelic homologous recombination. Indeed, human chromosomes contain up to 10% of repeated sequences dispersed throughout the genome. Non allelic homologous recombination between low copy number repeats is responsible for recombination-mediated chromosomal rearrangements included translocations, deletions, inversions and loss of heterozygosity both in mitosis and meiosis [185]. In fission yeast, a single collapsed fork can trigger translocation and genomic deletion in a recombination-dependent manner, showing that homologous recombination proteins allow fork restart but at the expense of genome instability [132,170]. Thus, a mechanism permitting the recruitment of recombination proteins at halted forks to stabilize them while preventing a strand exchange reaction might help to alleviate the double-edge sword effect of homologous recombination on genome maintenance. 

## 4. Concluding Remarks

The homologous recombination machinery acts as a replication fork-escort to support the efficiency of DNA replication by multiple mechanisms, including sealing of ssDNA gaps within newly replicated DNA, rebuilding of replisome at inactivated forks, and stabilization of halted forks. We propose that some fork-escort functions of the homologous recombination machinery might involve only the loading of recombination proteins onto single-stranded DNA without a strand exchange step. Activation of the DNA damage response has been observed in precancerous cells and during early-stages of malignancies, due to endogenous replication stress resulting from unbalanced DNA replication [186]. Initiation of DNA replication while all the conditions necessary for efficient DNA synthesis are not combined, such as a well-balanced dNTP pool, results ultimately in the slowdownof fork progression, fork collapse and breakage [187,188]. The completion and robustness of chromosome replication in such stress conditions are likely to rely on fork-escort mechanisms including checkpoint pathways and homologous recombination. Consistent with this, defects or malfunctions of the homologous recombination machinery are associated with cancer predisposition [189].The homologous recombination pathway becomes a drug target for anti-cancer therapy. Thus, it remains of high importance to decipher the molecular mechanisms by which the homologous recombination machinery ensures efficient DNA synthesis at defective replication forks in order to optimize inhibitors to target cell proliferation of cancers cells without inducing replication defects in healthy tissues.
